# Extreme genetic diversity in the lizard *Atlantolacerta andreanskyi* (Werner, 1929): A montane cryptic species complex

**DOI:** 10.1186/1471-2148-12-167

**Published:** 2012-09-04

**Authors:** Mafalda Barata, Salvador Carranza, D James Harris

**Affiliations:** 1 CIBIO, Centro de Investigação em Biodiversidade e Recursos Genéticos. Campus Agrário de Vairão, Vairão, 4485-661, Portugal; 2 Departamento de Biologia, Faculdade de Ciências da Universidade do Porto, Porto, 4099-002, Portugal; 3 Institute of Evolutionary Biology (CSIC-UPF), Barcelona, E-08003, Spain

**Keywords:** *Atlantolacerta andreanskyi*, Lacertidae, Mountain specialist, High Atlas Mountains, Phylogeography, Morocco

## Abstract

**Background:**

*Atlantolacerta andreanskyi* is an enigmatic lacertid lizard that, according to the most recent molecular analyses, belongs to the tribe Eremiadini, family Lacertidae. It is a mountain specialist, restricted to areas above 2400 m of the High Atlas Mountains of Morocco with apparently no connection between the different populations. In order to investigate its phylogeography, 92 specimens of *A. andreanskyi* were analyzed from eight different populations across the distribution range of the species for up to 1108 base pairs of mitochondrial DNA (*12S*, *ND4* and flanking *tRNA-His*) and 2585 base pairs of nuclear DNA including five loci (*PDC*, *ACM4*, *C-MOS*, *RAG1*, *MC1R*).

**Results:**

The results obtained with both concatenated and coalescent approaches and clustering methods, clearly show that all the populations analyzed present a very high level of genetic differentiation for the mitochondrial markers used and are also generally differentiated at the nuclear level.

**Conclusions:**

These results indicate that *A. andreanskyi* is an additional example of a montane species complex.

## Background

An emerging pattern among European biotas is that the accentuated environmental instability that occurred during the Pleistocene did not lead to increased speciation rates, with many species and populations originating during the Miocene and proceeding through the Quaternary [1,2]. In many species, population fragmentation was triggered by the beginning of the Messinian Salinity Crisis, a short (600 000 years) but crucial period that occurred between 5.9 and 5.3 Mya during which the Mediterranean Sea desiccated almost completely, producing a general and drastic increase in aridity around the Mediterranean Basin [3,4]. As a result of this increased aridity, forests continued to be replaced by more open and arid landscapes forcing the mesic species to retreat to the moister Atlantic-influenced areas and to the mountainous regions, leading to high speciation in some groups [5,6].

Various studies have attempted to unravel the different roles that the global aridification at the end of the Miocene and the Pleistocene glacial cycles have played in the diversity and distribution of European faunas [7]. However, little is known about the effects that these climatic changes had on species living further South, in the African continent. Recent assessments of central African chameleons have uncovered evidence of long-isolated evolutionary histories, with the survival of palaeoendemics leading to considerable diversity [8]. In general, reptiles are excellent model organisms to assess the relative role that the Pre-Quaternary and Quaternary major climatic events have played in the origin, evolution and distribution of species [9]. Available data from some herpetofauna indicate that a similar pattern to the neighboring Iberian Peninsula exists in North Africa, with deep lineages originating at the end of the Miocene (*Chalcides*[10], *Acanthodactylus*[11-13], *Podarcis*[2,14,15], *Saurodactylus*[16], *Ptyodactylus*[17], *Salamandra*[18], *Pleurodeles*[19]). However, the lack of informative nuclear markers in most of these studies may prevent the recovery of the true evolutionary history of the group [eg. [20,21], and makes it difficult to ascertain if these lineages correspond to species complexes or not. Since there is a strong likelihood of discordance between gene trees and species trees [22-24], information from different genetic markers (mitochondrial and nuclear) is thus necessary for delimiting evolutionary lineages, as well as for establishing phylogenetic relationships.

Despite being key concepts in the fields of systematic and evolutionary biology, recognizing and delimiting species are highly controversial issues ([e.g. [25,26]). Recognizing species is not only a taxonomic challenge, but is also essential for other biological disciplines such as biogeography, ecology and evolutionary biology [27], and has serious consequences for conservation biology and the design of effective conservation plans [28,29]. Delimiting species is also the first step towards discussing broader questions on evolution, biogeography, ecology or conservation. Recently, thanks to intellectual progress made in the field with the aim of identifying a common element among all the different species concepts, a single, more general, concept of species known as General Lineage Species Concept has been suggested [30]. This unified species concept emphasizes the common element found in many species concepts, which is that species are separately evolving lineages. Therefore, properties like reciprocal monophyly at one or multiple loci, phenotypic diagnosability, ecological distinctiveness, etc. are not part of the species concept but are used to assess the separation of lineages and to species delimitation [31]. This separation between species conceptualization and species delimitation and the proposal of a unified species concept has concentrated efforts in the development of new approaches for species delimitation, as for example with “integrative taxonomy” [32,33], among others]. Under this new approach, species delineation is regarded as an objective scientific process that results in a taxonomic hypothesis. Therefore, the level of confidence in the taxonomic hypothesis supported by several independent character sets is much higher than for species supported by only one character [34]. Such an integrative view is especially useful in the case of taxonomic groups that are morphologically conservative, where cryptic species have probably been overlooked [17,35,36].

Normally, high altitude species carry signatures of the expansion and contraction cycles occurred during glacial and interglacial periods [37-39]. Because of this, they are of particular interest to study historical responses to climate change, since they are adapted to a small window of environmental changes, and usually present low tolerance to high temperatures [40]. In Europe, high altitude species often seem to have persisted through glacial periods by short movements to lower altitudes rather than to the classic "southern refugia" of lowland species. In this way current ranges may primarily reflect postglacial expansions [41]. However, it is not clear if the same phenomenon occurs in African montane taxa.

**Figure 1:**
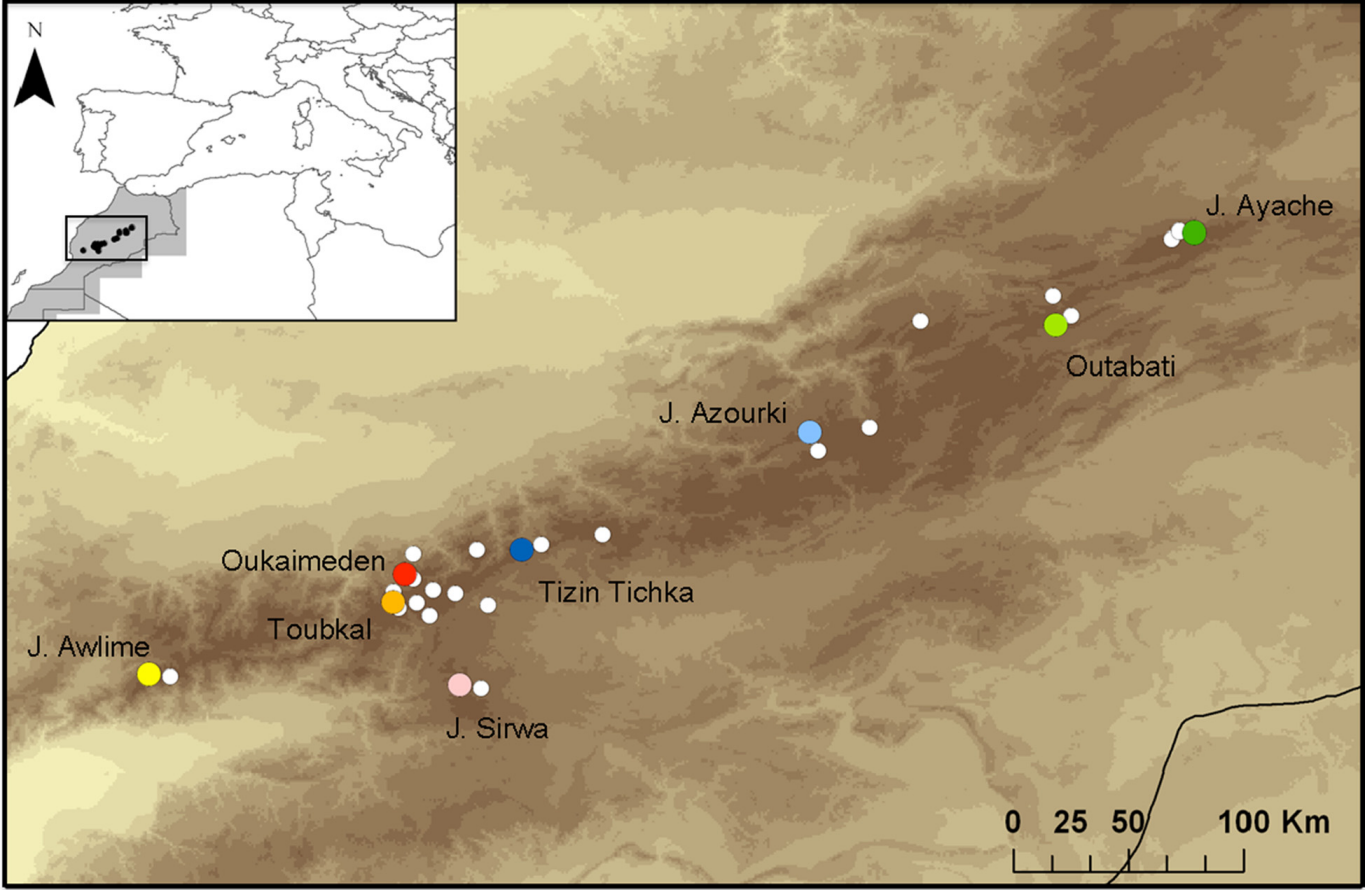
***Atlantolacerta andreanskyi *distribution map. ** The color dots represent the localities of the populations sampled for this work, J. Awlime (yellow), J. Sirwa (pink), Oukaimeden (red), Toubkal (orange), Tizin Tichka (dark blue), J. Azourki (light blue), Outabati (light green), and J. Ayache (dark green). The white dots represent the distributions of the species by Bons and Geniez [42].

*Atlantolacerta andreanskyi* (Werner, 1929) is a lacertid lizard endemic to the western and central parts of the High Atlas Mountains of Morocco. It is restricted to areas above 2400 m [42,43], where it is frequently found in the vicinity of small watercourses or plateaus in the top of the mountains that retain some water from rain or snowmelt. Habitat is normally screes and areas with boulders, meadows and, in particular, the base of cushion-like thorny plants in these places [42]; personal observation]. Although *A. andreanskyi* had initially been placed in several different genera within the subtribe Lacertini [44-48], recent phylogenetic analyses based on mitochondrial DNA and a combination of mitochondrial and nuclear markers [49,50] suggest that *A. andreanskyi* is a member of the subtribe Eremiadini, and apparently sister to the remaining Eremiadini. This position would conform to this species lacking the synapomorphies that characterize most other Eremiadini, namely a derived condition of the ulnar nerve and the presence of a fully developed armature in the hemipenis, which has folded lobes when retracted. It is also distinctive within the Eremiadini regarding the presence of enlarged masseteric scale [49]. Because of its phylogenetic position, without close relationship to any other genus of Eremiadini and its distinctive morphology it was recently placed in a new monotypic genus, *Atlantolacerta*[49]. *Atlantolacerta andreanskyi* is distributed across 440 Km (straight line) of mountainous terrain, with the different populations presenting an apparently disjunct distribution ([42,43]; see Figure 1). As with many montane species, the situation observed in *A. andreanskyi* is similar to an archipelago, with the different “islands” being represented by mountaintops disconnected due to areas of unsuitable habitat below 2400 m. As a result of this scenario, minimal gene flow is currently expected between the different populations; however, it is not known how the different climatic events occurred during the Miocene and Pleistocene have affected this species. Even though some aspects of the biology of *A. andreanskyi* are already well known [e.g. [51,52], the genetic structure of the different populations, as well as the relationships between the different populations have never been assessed before.

Therefore, in order to shed some light on the previous questions and attempt to assess the evolutionary history of the species and identify the number of lineages, we sampled the distribution area of the species and performed several combined phylogenetic reconstructions and clustering analyses, using both mtDNA and nuclear markers.

## Results

### Mitochondrial genealogies

A total of 1108 base pairs (bp) of concatenated mtDNA (*12S rRNA* 330 bp, *ND4* 592 bp and *tRNA-His* 186 bp) were obtained for 89 *A. andreanskyi*. The concatenated alignment of the ingroup sequences revealed 30 haplotypes (3 from Tizin Tichka, 7 from J. Ayache, 5 from J. Sirwa, 2 from Oukaimeden, 7 from J. Azourki, 2 from Outabati, 2 from Toubkal and 2 from J. Awlime) and contained 241 variable sites, of which 232 were parsimony informative.

**Figure 2:**
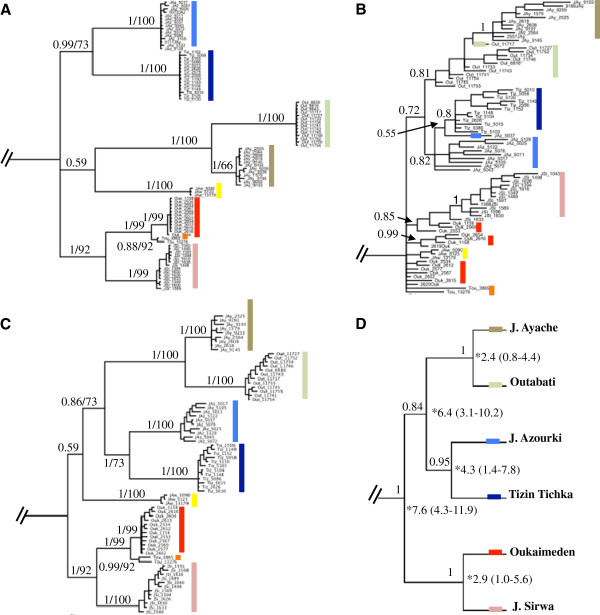
**Trees resulting from partitioned Bayesian analysis.** (**A**) mitochondrial DNA tree (*12S*, *ND4* and flanking *tRNA-His*), (**B**) nuclear concatenated tree (*RAG1*, *ACM4*, *MC1R*, *PDC* and *C-MOS*), (**C**) Concatenated tree from the combined mitochondrial and nuclear DNA data. The partitions used the models described in the text. Bayesian posterior probabilities (0–1) and bootstrap values (> 50%) for ML (1–100) are indicated near the branches, (**D**) Species tree from mitochondrial and nuclear DNA data from the Bayesian Inference of Species Trees (STARBEAST). Clade posterior probabilities are shown to the left of the nodes, and divergence times and 95% intervals (calculated in BEAST using only *ND4 + tRNA-His*), to the right of the nodes. The trees were rooted using *Podarcis bocagei*, *P. hispanica* and *P. carbonelli*. The colors represent the different populations.

Analyses of the concatenated mtDNA data were mostly congruent (Figure 2A). Seven well-supported lineages were recovered from these analyses (pp > 0.95 and BP > 70%), corresponding to the populations from J. Awlime, J. Sirwa, Tizin Tichka, J. Azourki, Outabati, J. Ayache, and Oukaimeden and nearby Toubkal that were grouped together. Regarding the relationships among these clades, we could distinguish three main groups, Oukaimeden and Toubkal with J. Sirwa from the southern end of the distribution range; J. Ayache with Outabati from the northern distribution, and Tizin Tichka with J. Azourki from the central distribution range. The population from J. Awlime, from the extreme South of the range, is a genetically distinct lineage related to the northern group, although, both ML and BI analysis weakly support this topology (see Figure 2A).

All the populations present a low level of diversity in the mitochondrial DNA (uncorrected genetic distances 0–0.5% for the *ND4 + tRNA-His* and 0 – 0.2% for the *12S*; see Table 1), and a very high level of genetic divergence between populations (5.5 – 16.5% in the *ND4 + tRNA-His* and 2.5 – 6.6% in the *12S*).

**Table 1:** Genetic distances and divergence time estimate between populations

**A**								
**Pop p-distance (%) 12S, ND4**	**Tizin Tichka**	**Oukaimeden**	**J. Sirwa**	**J. Ayache**	**Outabati**	**J. Azourki**	**Toubkal**	**J. Awlime**
	0.1	0.4	0.3	0.5	0.2	0	0.4	0.1
**Tizin Tichka**		13.1	12.7	14.5	10.5	15.3	12.9	13.6
0								
**Oukaimeden**	4		7.7	15	13.2	16.1	1.7	13.2
0								
**J. Sirwa**	4.2	2.8		16.1	12.7	16.5	7.5	11.6
0.2								
**J. Ayache**	5.4	5.7	4.8		12.7	5.5	14.4	14.1
0.1								
**Outabati**	4.3	4.3	3.8	6.6		14.2	13.2	13.1
0.1								
**J. Azourki**	5.4	5.7	4.2	1.6	6		16	14
0								
**Toubkal**	3.7	0.3	2.5	5.4	4	5.4		12.6
0								
**J. Awlime**	4	4.7	4.5	5.1	6.4	5	4.3	
0								
**B**						**C**		
**Pop p-distance (%) 12S and ND4**	**J. Awlime**							
	**JAy + Out**	**Tiz + JAz**		**Ouk + JSi + Tou**		**Beast Ma (95% HPD)**	***ND4***	
	0.9	2.3	0	1.5				
JAy + JAz		13.7	13.4	14.6		**North - South**	7.6 (4.3-11.9)	
2.9						**Jaw - Ouk**	5.6 (2.5-9.7)	
Tiz + Ou	5.9		12.9	12.4		**JAz + JAy - Out + Tiz**	6.4 (3.1-10.2)	
5.2						**Ouk - JSi**	2.9 (1.0-5.6)	
J. Awlime	5	5.2		12.2		**Out - Tiz**	4.3 (1.4-7.8)	
0.1						**JAz - JAy**	2.4 (0.8-4.4)	
Ouk + JSi + Tou	5.1	4.1	4.6			**Ouk - Tou**	0.5 (0.1-1.2)	
0.4								

(A) Genetic distance (*12S* and *ND4 + tRNA-His*) between all the populations and (B) between main groups; and (C) divergence time estimates, calculated using BEAST with *ND4* and *tRNA-His*. The the diversity of each population is below the population's names.

### Nuclear genealogies

A total of 77 specimens of *A. andreanskyi* were sequenced for five nuclear genes. The *ACM4* was 447 bp long, presenting 47 haplotypes and 34 polymorphic sites, 33 of them parsimony informative; *C-MOS* was 534 bp long, with 32 haplotypes and 21 polymorphic sites, all of them parsimony informative; *MC1R* was 635 bp long, with 57 haplotypes and 36 variable sites, 35 of them parsimony informative; *PDC* was 441 bp long, with 60 haplotypes and 29 variable sites, 26 of them parsimony informative; *RAG1* was 528 bp long, with 38 haplotypes and 19 variable sites, 18 of them parsimony informative.

The differences in the genetic distances between the lineages are congruent with the geographic distance between them, supporting the grouping of the lineages in three main groups as seen in the analysis of mitochondrial sequences.

The concatenated analyses of the 5 unphased nuclear markers are congruent with the results obtained in the mitochondrial DNA tree, although with some differences (Figure 2B). Despite recovering the three main groups observed in the mtDNA analysis, according to the nuclear markers the J. Awlime population is not sister to the northernmost populations but branches off inside a polytomy with the westernmost lineages at the base of the tree. It is possible to distinguish some of the lineages, although in some cases they are not monophyletic. The J. Ayache population is monophyletic but makes Outabati paraphyletic. The same happens with Tizin Tichka, which makes the population from J. Azourki paraphyletic. The population from Oukaimeden is polyphyletic.

### Concatenated analysis (mtDNA and nDNA)

The results of the ML and BI analyses of the mtDNA and nDNA (Figure 2C) support the same seven lineages as recovered in the mitochondrial analysis, although in this case J. Awlime is sister to the central and northern lineages (Tizin Tichka, J. Azourki, Outabati, and J. Ayache) instead of being sister to only the northernmost lineages (Figure 2A). As in the mtDNA analysis (Figure 2A), the relationship of J. Awlime with the central and northern lineages is very poorly supported. This result was expected, given the higher resolving power of the mtDNA that contributed with 241 variable sites versus the 150 from the nDNA.

### Nuclear networks

**Figure 3:**
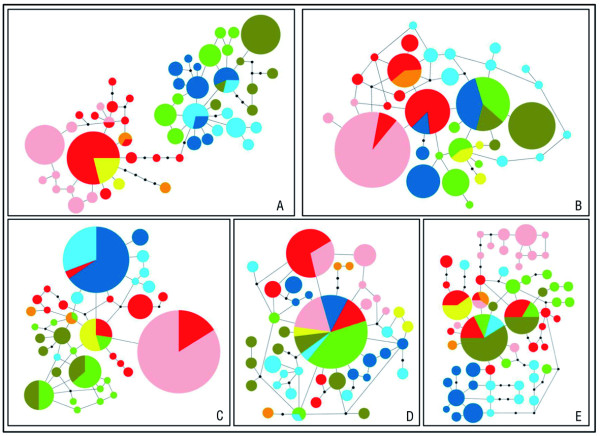
**Parsimony networks corresponding to *MC1R *(A), *RAG1 *(B), *C-MOS *(C), *ACM4 *(D) and *PDC *(E) nDNA sequence variation from all the populations.** The colors used were the same as the used in the map (Figure 1) and trees (Figure 2), J. Awlime (yellow), Toubkal (orange), Oukaimeden (red), J. Sirwa (pink), Tizin Tichka (dark blue), J. Azourki (light blue), Outabati (light green), and J. Ayache (dark green). Lines represent a mutation step, circles represent haplotypes and dots missing haplotypes. The size of the circles is proportional to the number of alleles.

As show in Figure 3 and Table 2, there is a moderate degree of haplotype sharing between populations, with most of them lacking private alleles for the nuclear genes analyzed.

**Table 2:** Percentage of private alleles in all the populations and for each nuclear locus

**Private Alleles (%)**	***MCIR***	***RAGI***	***C-MOS***	***ACM4***	***PDC***
J. Awlime	33	50	0	67	0
J. Sirwa	96	12	0	42	92
Toubkal	50	0	50	100	50
Oukaimeden	41	33	70	29	57
Tizin Tichka	75	59	23	71	100
J. Azourki	60	100	60	84	90
Outabati	100	54	43	9	83
J. Ayache	92	85	57	80	20

### Clustering analysis and individual assignment

**Figure 4:**
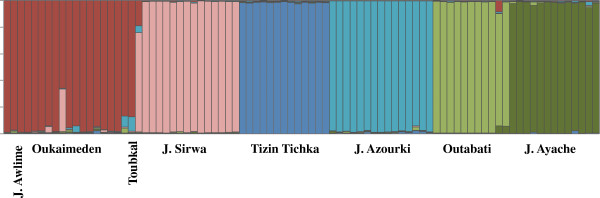
**Population structure estimation.** Each individual is represented by a thin vertical line, which is partitioned into K colored segments that represent the individual’s estimated membership fractions in K clusters. The bigger vertical divisions separate individuals from different populations. Populations are labeled below the figure. The colors used are the same used in Figure 1 and Figure 2.

In our study, the obtained K differs with the combination between the ancestry model and the allele frequency model. When combined the No Admixture Model (ancestry model) with the Allele Frequencies Independent Model (allele frequency model) the best resulting K values where for K = 3: South (Oukaimeden, J. Sirwa, Toubkal and J. Awlime), center (Tizin Tichka and J. Azourki) and North (Outabati and J. Ayache) groups. With the other three combinations between the models the best result were for K = 6: J. Sirwa, Tizin Tichka, J. Azourki, Outabati, J. Ayache, and a group formed by Oukaimeden, Toubkal and J. Awlime (Figure 4).

### Species tree and divergence time estimates

The results of the clustering analysis with K = 6 were used to define the species for the species tree analysis in STARBEAST. The tree inferred with information from mitochondrial and nuclear markers (phased) (figure 2D) recovered the same topology as in Figure 2C, with all the relationships between the lineages supported by previous analyses.

The divergence time estimates were calculated for the six populations (Table 1). High effective sample sizes were observed for all parameters in all BEAST analysis (posterior ESS values > 1000 for all four analyses) and assessment of convergence statistics in Tracer indicated that all analyses had converged. Maximum clade credibility tree for *ND4 + tRNA-His* was identical in topology to those produced by Bayesian and ML analyses. According to the inferred dates resulted from BEAST (Figure 2D), the two main mitochondrial lineages of *A. andreanskyi* (South versus central and North) split approximately 7.6 Ma (95% high posterior density (HPD) interval 4.3-11.9 Ma). The populations that are grouped in the three main clades (South, central and North) split approximately at the same time, being Tizin Tichka and J. Azourki the first to split at about 4.3 Ma (1.4-7.8), followed by Oukaimeden and J. Sirwa 2.9 Ma (1–5.6), and Outabati and J. Ayache 2.4 Ma (0.8-4.4). Tizin Tichka and J. Azourki diverged from Outabati and J. Ayache approximately 6.4 Ma (3.1-10.2).

## Discussion

### Extreme mtDNA diversity in *A. andreanskyi*

Several recently published analyses of North African herpetofauna have revealed high levels of endemism and cryptic species [12,14,15,17]. In this analysis, the surprising result was the extreme diversity of mitochondrial DNA found between almost all the populations analyzed The genetic differentiation observed between populations (2.8% - 6.6% in *12S* and 5.5% - 16.5% in *ND4 + tRNA-His*) is similar and, in some cases, higher than the divergence found between *Iberolacerta* species (between 7.4% and 8.2% in the *cytochromeb* gene, [53]), a lacertid genus with most of its species occurring in the mountains of the Iberian Peninsula [41,54]. Initially considered one species, there are now seven recognized species of *Iberolacerta* in the Iberian Peninsula. Genetic differentiation between these species is lower than between the different populations of *A. andreanskyi*.

Although the mitochondrial phylogeny supports the existence of seven distinct groups, the clustering analysis only supports the existence of six lineages (J. Sirwa, Tizin Tichka, J. Azourki, Outabati, J. Ayache and a lineage formed by Oukaimeden, Toubkal and J. Awlime). Toubkal samples were always part of the same lineage as Oukaimeden, although, they show some divergence at least at the mitochondrial DNA level (1.7% in *ND4 + tRNA-His* and 0.3% in *12S*). This is not unexpected, as these populations are geographically very close and are part of the High Atlas Mountains, where interconnectivity between populations could occur. The mitochondrial phylogenetic analyses supported the existence of a seventh isolated lineage, J. Awlime, however clustering analysis and the nuclear phylogeny did not support the distinctiveness of this population, possibly because of the small sampling size. Unfortunately, despite multiple attempts to sample in this remote region, only three individuals were captured. The analyses also could not recover the genetic relationship between J. Awlime and the other populations, because its position in the trees fluctuated between the two main groups (North and South), without support in any of the trees.

### Non-reciprocal monophyly in nuclear markers and species delimitation

In the phylogenetic analyses of the concatenated nuclear loci, some of the lineages supported by mtDNA data were not monophyletic. This was observed only between the geographically closest lineages, as in the case of Oukaimeden and J. Sirwa; Tizin Tichka and J. Azourki; and Outabati and J. Ayache, that presumably were in contact more recently than the others. This may be due to the larger effective population size of the nuclear DNA compared to the mitochondrial DNA and the consequent stronger effect of the incomplete lineage sorting at each single nuclear loci [55]. Additionally, the slow evolutionary rate of some of these markers may be a factor. The conjugation of these two effects probably explains the absence of concordance in the single nuclear gene trees (not show), although the same general topology was recovered in the concatenated nuclear phylogeny. Reciprocal monophyly is one of the primary criteria to delimit species [31,56]. Although it is possible to delimit species without observing monophyly in gene trees, since a considerable amount of time must pass after the beginning of divergence of species until they show reciprocal monophyly at a sample of multiple loci [57,58]. Pinho *et al.*[59] have shown that *Podarcis* from the Iberian Peninsula and North Africa have a similar pattern (between mtDNA and nuclear) but in a smaller time window and using faster evolving nuclear loci and, in contrast to our case, some populations are in contact.

Although we are aware that the determination of K, in STRUCTURE, is only an *ad hoc* guide to describe consistence between models and the data [60], the program has been commonly used for this end [61]. Several methods based on Bayesian clustering have been developed [62-64], however, STRUCTURE is the most widely used, and various studies show its efficiency in assigning individuals to their population of origin [65-68] and its ability to construct an appropriate clustering hypothesis [61]. However, in the present example the analysis was limited because it was based only in haplotype information. The obtained K differ with the combination model used, but in most of the combinations the analysis supports a K = 6 corresponding to the geographical populations and to the results recovered by the other analyses. This analysis also placed the samples from the J. Awlime population together with the Oukaimeden lineage, possibly due to the limited haplotype sampling. Similarly, the concatenated phylogenetic tree, based on all the genes, supports the existence of 7 lineages giving once more a low support to the relationship between J. Awlime and the other lineages.

The networks of the individual nuclear loci show high percentage of private alleles in some of the lineages, which fluctuate depending on the gene.

### Dating the trees

All the lineages are grouped in two main clusters, the northern group composed by J. Ayache, Outabati, J. Azourki and Tizin Tichka; and the southern group that includes Oukaimeden and J. Sirwa. The divergence obtained for these two lineages was around 7.6 Mya, (4.3-11.9), which coincides approximately with the time of the final closing of the Rifian Strait (7.2 Mya; [3]). During the Miocene, tectonic activity in the region was intense and included the uplift of the Atlas Mountains that occurred around 9.0 Mya [69,70]. It was more or less at the same time that *Podarcis* invaded North Africa (7.5 ± 1.2 Mya, [2]) and the Iberian clade of *Iberolacerta* started to fragment (6.1 Mya, [1]). The split of the six lineages must have occurred later, probably during the Quaternary Glaciations (4.3 ± 3; 2.4 ± 2; 2.9 ± 2 Mya). However, the confidence intervals obtained were very large, increasing the time window for the events and the associated error. Determination of the time of the speciation events is important to understand the evolutionary biogeography of species [71]. However, it is difficult to estimate ages in phylogenies without several sources of error. Clearly the lineages of *A. andreanskyi* are pre-Pleistocenic and, as found in Central African chameleons [8] can be considered paleoendemics. However, without better calibration points it is difficult to date the split of the lineages more precisely than this.

## Conclusions

Phylogeographic assessments of several taxa in northwest Africa have indicated the presence of cryptic diversity in organisms ranging from scorpions [72] to mammals [73], and reptiles are not an exception [e.g. [11,17,74]. What is exceptional in the case of *A. andreanskyi* are the high levels of mitochondrial divergence between almost every sampled populations, ranging from 5.5 up to 16.5% (*ND4 + tRNA-His*) between populations separated by low geographic distances (for example just 60 Km between Oukaimeden and J. Sirwa and 45 Km between Oukaimeden and Tizin Tichka). Six of the eight analyzed populations are highly distinct based on both mtDNA and multiple nuclear markers. This raises the issue not of whether *A. andreanskyi* is a species complex, but just how many species may occur within the group. Presumably, far more than the six possible species identified in this study, since, probably, many populations remain unsampled. However, preliminary morphological analyses suggest that all the different populations included in the present study are very homogeneous (unpublished data). This may imply the presence of cryptic diversity, but definitive conclusions should wait until a complete morphological study is carried out (work in progress).

Current models of reptiles species accessed for the region indicate low levels of diversity across much of the High Atlas Mountains [75]. Indeed only a few species are recorded at altitudes above 2000 m; typically *A. andreanskyi*, *Quedenfeldtia* species (*Q. trachyblepharus* and *Q. moerens*), *Chalcides montanus* and *Vipera monticola* [e.g. [42,76]. However, the finding of high genetic diversity in *A. andreanskyi* indicates that unidentified lineages occur, and that the other high mountain species should also be assessed as possible cryptic species candidates. Our results are also essential from a conservation point of view, as many forms may actually have smaller ranges than currently thought, and small isolated populations on high mountains have been identified as those of high concern under typical global warming scenarios [77]. Given these results it is necessary to increase the sampling in order to understand the relationship of J. Awlime with the other populations and try to find new populations. Furthermore it is very important to conduct a through morphological study to determine if there is phenotypic variation, and then to revise the taxonomy of the genus *Atlantolacerta*.

## Methods

### Species concept and integrative approach

Although the present study does not include a taxonomic revision of the genus *Atlantolacerta*, like many other works in which some of the authors of the present manuscript have participated [35,78,79], we advocate for the use of the General Lineage Species Concept proposed by de Queiroz [30]. Two lines of evidence have been defined on the basis of alleged independence of their respective datasets: mitochondrial DNA and nuclear DNA. In the present study, we have decided to retain as “putative species” only these lineages that were recovered as monophyletic in the phylogenetic analysis of the mtDNA data and that were supported by the analysis of the nDNA using STRUCTURE v.2.3.2 [60]. Within the framework of an integrative approach, and pending the inclusion of morphological data, this would correspond to Integration by total congruence (ITC). However, it is important to take into account that in the absence of a thorough morphological analysis we do not consider the molecular data presented here enough to revise the taxonomy of the genus *Atlantolacerta.*

### DNA extraction, amplification, and sequencing

A total of 92 individuals from eight different populations distributed across the entire range of *Atlantolacerta andreanskyi* were sampled for this study: 14 from Oukaimeden, 15 from Tizin Tichka, 14 from Jebel Ayache, 15 from Jebel Azourki, 14 from Outabati, 15 from Jebel Sirwa and 2 from Toubkal and 3 from J. Awlime (Figure 1 and Table 3). Specimens were caught by hand, identified on the basis of external features, measured and photographed for later morphological studies. Tail tips where collected and stored in 96% ethanol, after which individuals were released in the same place where they were caught.

**Table 3:** Samples used in the work with localities (GPS coordinates; WGS84 coordinate system) and GenBank accession numbers for all the sequenced genes

						**GenBank Acession codes**						
**Specimen code**	**Alleles**	**Population**	**Latitude**	**Longitude**	**Altitude**	***12S***	***ND4 + tRNA-His***	***PDC***	***ACM4***	***C-MOS***	***MC1R***	***RAG1***
1152	1152a	Tizin Tichka (Tiz)	31.30077	−7.40984	2800	JX462053	JX462189	JX461527	JX461879	JX485185	JX461693	JX461351
	1152b							JX461528	JX461880	JX485186	JX461694	JX461352
1149	1149a	Tizin Tichka (Tiz)	31.30077	−7.40984	2800	JX462062	JX462194	JX461523	JX461875	JX485189	JX461689	JX461349
	1149b							JX461524	JX461876	JX485190	JX461690	JX461350
1148	1148a	Tizin Tichka (Tiz)	31.30077	−7.40984	2800	JX462064	JX462195	JX461521	JX461873	JX485191	JX461687	JX461347
	1148b							JX461522	JX461874	JX485192	JX461688	JX461348
1150	1150	Tizin Tichka (Tiz)	31.30077	−7.40984	2800	JX462061	JX462191	…	…	…	…	…
2556	2556a	Tizin Tichka (Tiz)	31.30077	−7.40984	2800	JX462060	JX462192	JX461593	JX461947	JX485195	JX461947	JX461417
	2556b							JX461594	JX461948	JX485196	JX461948	JX491418
2578	2578	Tizin Tichka (Tiz)	31.30077	−7.40984	2800	JX462059	JX462193	…	…	…	…	…
2626	2626a	Tizin Tichka (Tiz)	31.30077	−7.40984	2800	JX462058	JX462190	JX461625	JX461979	JX485193	JX461793	JX461447
	2626b							JX461626	JX461980	JX485194	JX461794	JX461448
5058	5058a	Tizin Tichka (Tiz)	31.30077	−7.40984	2800	JX462054	JX462196	JX461643	JX461999	JX485205	JX461815	JX461469
	5058b							JX461644	JX462000	JX485206	JX461816	JX461470
5010	5010a	Tizin Tichka (Tiz)	31.30077	−7.40984	2800	JX462065	JX462203	JX461629	JX461983	JX485199	JX461799	JX461453
	5010b							JX461630	JX461984	JX485200	JX461800	JX461454
5126	5126	Tizin Tichka (Tiz)	31.30077	−7.40984	2800	JX462066	JX462197	…	…	…	…	…
5086	5086a	Tizin Tichka (Tiz)	31.30077	−7.40984	2800	JX462056	JX462198	JX461649	JX462009	JX485197	JX461825	JX461479
	5086b							JX461650	JX462010	JX485198	JX461826	JX461480
5103	5103a	Tizin Tichka (Tiz)	31.30077	−7.40984	2800	JX462055	JX462202	JX461655	JX462015	JX485207	JX461831	JX461483
	5103b							JX461656	JX462016	JX485208	JX461832	JX461484
5104	5104a	Tizin Tichka (Tiz)	31.30077	−7.40984	2800	JX462063	JX462199	JX461657	JX462017	JX485209	JX461833	JX461485
	5104b							JX461658	JX462018	JX485210	JX461834	JX461486
5015	5015a	Tizin Tichka (Tiz)	31.30077	−7.40984	2800	JX462057	JX462200	JX461633	JX461987	JX485203	JX461803	JX46147
	5015b							JX461634	JX461988	JX485204	JX461804	JX46148
5130	5130a	Tizin Tichka (Tiz)	31.30077	−7.40984	2800	JX462067	JX462201	JX461667	JX462031	JX485201	JX461847	JX461497
	5130b							JX461668	JX462032	JX485202	JX461848	JX461498
1040	1040a	Jebel Sirwa (JSi)	30.77671	−7.65299	2561	JX462083	JX462153	JX461519	JX461871	JX485240	JX461685	JX461345
	1040b							JX461520	JX461872	JX485241	JX461686	JX461346
1349	1349a	Jebel Sirwa (JSi)	30.77671	−7.65299	2561	JX462084	JX462150	JX461557	JX461911	JX485141	JX461725	JX461383
	1349b							JX461558	JX461912	JX485142	JX461726	JX461384
1394	1394a	Jebel Sirwa (JSi)	30.77671	−7.65299	2561	JX462085	JX462147	JX461559	JX461913	JX485244	JX461726	JX461384
	1394b							JX461560	JX461914	JX485245	JX461727	JX461385
1489	1489a	Jebel Sirwa (JSi)	30.77671	−7.65299	2561	JX462086	JX462152	JX461561	JX461915	JX485246	JX461729	JX461387
	1489b							JX461562	JX461916	JX485247	JX461730	JX461388
1498	1498a	Jebel Sirwa (JSi)	30.77671	−7.65299	2561	JX462087	JX462151	JX461563	JX461917	JX485248	JX461732	JX461389
	1498b							JX461564	JX461918	JX485249	JX461733	JX461390
1598	1598a	Jebel Sirwa (JSi)	30.77671	−7.65299	2561	JX462158	JX462158	JX461573	JX461927	JX485256	JX461741	JX461399
	1598b							JX461574	JX461928	JX485257	JX461742	JX461340
1633	1633a	Jebel Sirwa (JSi)	30.77671	−7.65299	2561	JX462096	JX462148	JX461583	JX461937	JX485264	JX461751	JX461409
	1633b							JX461584	JX461938	JX485265	JX461752	JX461410
1638	1638	Jebel Sirwa (JSi)	30.77671	−7.65299	2561	JX462097	JX462159	…	…	…	…	…
1638	1588a	Jebel Sirwa (JSi)	30.77671	−7.65299	2561	…	…	JX461567	JX461921	JX485250	JX461735	JX461393
	1588b							JX461568	JX461922	JX485251	JX461736	JX461394
1626	1626a	Jebel Sirwa (JSi)	30.77671	−7.65299	2561	JX462160	JX462160	JX461579	JX461933	JX485260	JX461747	JX461405
	1626b							JX461580	JX461934	JX485261	JX461748	JX461406
1616	1616a	Jebel Sirwa (JSi)	30.77671	−7.65299	2561	JX462154	JX462154	JX461577	JX461931	JX485258	JX461745	JX461403
	1616b							JX461578	JX461932	JX485259	JX461746	JX461404
1609	1609	Jebel Sirwa (JSi)	30.77671	−7.65299	2561	JX462155	JX462155	…	…	…	…	…
1589	1589a	Jebel Sirwa (JSi)	30.77671	−7.65299	2561	JX462090	JX462156	JX461569	JX461923	JX485252	JX461737	JX461395
	1589b							JX461570	JX461924	JX485253	JX461738	JX461396
1630	1630a	Jebel Sirwa (JSi)	30.77671	−7.65299	2561	JX462088	JX462149	JX461581	JX461935	JX485262	JX461749	JX461407
	1630b							JX461582	JX461936	JX485263	JX461750	JX461408
1591	1591a	Jebel Sirwa (JSi)	30.77671	−7.65299	2561	JX462157	JX462157	JX461571	JX461925	JX485254	JX461739	JX461397
	1591b							JX461572	JX461926	JX485255	JX461740	JX461398
1158	1158a	Oukaimeden (Ouk)	31.20426	−7.86705	2600	JX462069	JX462162	JX461531	JX461883	JX485211	JX461697	JX461355
	1158b							JX461532	JX461884	JX485212	JX461698	JX461356
1154	1154a	Oukaimeden (Ouk)	31.20426	−7.86705	2600	JX462068	JX462161	JX461529	JX461881	JX485214	JX461695	JX461353
	1154b							JX461530	JX461882	JX485215	JX461696	JX461354
2534	2534a	Oukaimeden (Ouk)	31.20426	−7.86705	2600	JX462070	JX462163	JX461587	JX461941	JX485220	JX461755	JX461411
	2534b							JX461588	JX461942	JX485221	JX461756	JX461412
2553	2553a	Oukaimeden (Ouk)	31.20426	−7.86705	2600	JX462071	JX462164	JX461591	JX461945	JX485218	JX461759	JX461415
	2553b							JX461592	JX461946	JX485219	JX461760	JX461416
2619	2619a	Oukaimeden (Ouk)	31.20426	−7.86705	2600	…	…	JX461621	JX461975	JX485238	JX461789	JX461443
	2619b							JX461622	JX461976	JX485239	JX461790	JX461444
2620	2620a	Oukaimeden (Ouk)	31.20426	−7.86705	2600	…	…	JX461623	JX461977	JX485236	JX461791	JX461445
	2620b							JX461624	JX461978	JX485237	JX461792	JX461446
2577	2577a	Oukaimeden (Ouk)	31.20426	−7.86705	2600	JX462074	JX462167	JX461603	JX461957	JX485216	JX461771	JX461427
	2577b							JX461604	JX461958	JX485217	JX461772	JX461428
2567	2567a	Oukaimeden (Ouk)	31.20426	−7.86705	2600	JX462072	JX462165	JX461599	JX461953	JX485222	JX461767	JX461423
	2567b							JX461600	JX461954	JX485223	JX461768	JX461424
2569	2569a	Oukaimeden (Ouk)	31.20426	−7.86705	2600	JX462073	JX462166	JX461601	JX461955	JX485234	JX461769	JX461425
	2569b							JX461602	JX461956	JX485235	JX461770	JX461426
2602	2602a	Oukaimeden (Ouk)	31.20426	−7.86705	2600	JX462075	JX462168	JX461607	JX461961	JX485232	JX461775	JX461429
	2602b							JX461608	JX461962	JX485233	JX461776	JX461430
2604	2604a	Oukaimeden (Ouk)	31.20426	−7.86705	2600	JX462076	JX462169	JX461609	JX461963	JX485230	JX461777	JX461430
	2604b							JX461610	JX461964	JX485231	JX461778	JX461431
2612	2612a	Oukaimeden (Ouk)	31.20426	−7.86705	2600	JX462077	JX462170	JX461613	JX461967	JX485224	JX461781	JX461435
	2612b							JX461614	JX461968	JX485225	JX461782	JX461436
2615	2615a	Oukaimeden (Ouk)	31.20426	−7.86705	2600	JX462078	JX462171	JX461615	JX461969	JX485228	JX461783	JX461437
	2615b							JX461616	JX461970	JX485229	JX461784	JX461438
2616	2615a	Oukaimeden (Ouk)	31.20426	−7.86705	2600	JX462079	JX462172	JX461617	JX461971	JX485226	JX461785	JX461439
	2615b							JX461618	JX461972	JX485227	JX461786	JX461440
1579	1579a	Jebel Ayache (Jay)	32.53671	−4.79110	3043	JX462098	JX462178	JX461565	JX461919	JX485276	JX461733	JX461391
	1579b							JX461566	JX461920	JX485277	JX461734	JX461392
2552	2552a	Jebel Ayache (Jay)	32.53671	−4.79110	3043	JX462099	JX462177	JX461589	JX461943	JX485278	JX461757	JX461413
	2552b							JX461590	JX461944	JX485279	JX461758	JX461414
2564	2694a	Jebel Ayache (Jay)	32.53671	−4.79110	3043	JX462101	JX462176	JX461597	JX461951	JX485272	JX461765	JX461421
	2694b							JX461598	JX461952	JX485273	JX461766	JX461422
2608	2608a	Jebel Ayache (Jay)	32.53671	−4.79110	3043	JX462102	JX462175	JX461611	JX461965	JX485270	JX461779	JX461433
	2608b							JX461612	JX461966	JX485271	JX461780	JX461434
2618	2618a	Jebel Ayache (Jay)	32.53671	−4.79110	3043	JX462103	JX462174	JX461619	JX461973	JX485268	JX461787	JX461441
	2618b							JX461620	JX461974	JX485269	JX461788	JX461442
9189	9189a	Jebel Ayache (Jay)	32.53671	−4.79110	3043	…	…	JX461675	JX462043	JX485282	JX461859	JX461509
	9189b							JX461676	JX462044	JX485283	JX461860	JX461510
9199	9199	Jebel Ayache (Jay)	32.53671	−4.79110	3043	JX462108	JX462183	…	…	…	…	…
9255	9255a	Jebel Ayache (Jay)	32.53671	−4.79110	3043	JX462110	JX462179	JX461681	JX462051	JX485290	JX461867	JX461515
	9255b							JX461682	JX462052	JX485291	JX461868	JX461516
9209	9209	Jebel Ayache (Jay)	32.53671	−4.79110	3043	JX462109	JX462184	…	…	…	…	…
9191	9191a	Jebel Ayache (Jay)	32.53671	−4.79110	3043	JX462106	JX462181	JX461677	JX462045	JX485292	JX461861	JX461511
	9191b							JX461678	JX462046	JX485293	JX461862	JX461512
9336	9336	Jebel Ayache (Jay)	32.53671	−4.79110	3043	JX462111	JX462182	…	…	…	…	…
9193	9193a	Jebel Ayache (Jay)	32.53671	−4.79110	3043	JX462107	JX462188	JX461679	JX462047	JX485288	JX461863	JX461513
	9193b							JX461680	JX462048	JX485289	JX461864	JX461514
2557	2557a	Jebel Ayache (Jay)	32.53671	−4.79110	3043	…	…	JX461595	JX461949	JX485274	JX461763	JX461419
	2557b							JX461596	JX461950	JX485275	JX461764	JX461420
9145	9145a	Jebel Ayache (Jay)	32.53671	−4.79110	3043	JX462104	JX462185	JX461673	JX462041	JX485286	JX461857	JX461507
	9145b							JX461674	JX462042	JX485287	JX461858	JX461508
5076	5076a	Jebel Azourki (Jaz)	31.75847	−6.28826	2789	JX462120	JX462206	JX461647	JX462005	JX485296	JX461821	JX461475
	5076b							JX461648	JX462006	JX485297	JX461822	JX461476
5128	5128a	Jebel Azourki (Jaz)	31.75847	−6.28826	2789	JX462126	JX462207	JX461665	JX462029	JX485298	JX461845	JX461495
	5128b							JX461667	JX462030	JX485299	JX461846	JX461496
5091	5091	Jebel Azourki (Jaz)	31.75847	−6.28826	2789	JX462122	JX462208	…	…	…	…	…
5017	5017a	Jebel Azourki (Jaz)	31.75847	−6.28826	2789	JX462113	JX462209	JX461635	JX461989	JX485308	JX461805	JX461459
	5071b							JX461636	JX461990	JX485309	JX461806	JX461460
5122	5122a	Jebel Azourki (Jaz)	31.75847	−6.28826	2789	JX462125	JX462210	JX461661	JX462023	JX485300	JX461839	JX461491
	5122b							JX461662	JX462024	JX485301	JX461840	JX461492
5105	5105a	Jebel Azourki (Jaz)	31.75847	−6.28826	2789	JX462123	JX462211	JX461659	JX462019	JX485302	JX461835	JX461487
	5105b							JX461660	JX462020	JX485303	JX461836	JX461488
5072	5072a	Jebel Azourki (Jaz)	31.75847	−6.28826	2789	JX462118	JX462221	JX461645	JX462001	JX485304	JX461817	JX461471
	5072b							JX461646	JX462002	JX485305	JX461818	JX461472
5037	5037a	Jebel Azourki (Jaz)	31.75847	−6.28826	2789	JX462116	JX462213	JX461639	JX461995	JX485322	JX461811	JX461465
	5037b							JX461640	JX461996	JX485323	JX461812	JX461466
5011	5011a	Jebel Azourki (Jaz)	31.75847	−6.28826	2789	JX462112	JX462204	JX461631	JX461985	JX485312	JX461801	JX461455
	5011b							JX461632	JX461986	JX485313	JX461802	JX461456
5034	5034	Jebel Azourki (Jaz)	31.75847	−6.28826	2789	JX462115	JX462205	…	…	…	…	…
5080	5080	Jebel Azourki (Jaz)	31.75847	−6.28826	2789	JX462121	JX462216	…	…	…	…	…
5025	5025a	Jebel Azourki (Jaz)	31.75847	−6.28826	2789	JX462114	JX462214	JX461637	JX461991	JX485314	JX461807	JX461461
	5025b							JX461638	JX461992	JX485315	JX461808	JX461462
5043	5043a	Jebel Azourki (Jaz)	31.75847	−6.28826	2789	JX462117	JX462218	JX461641	JX461997	JX485324	JX461813	JX461467
	5034b							JX461641	JX461997	JX485324	JX461813	JX461467
5073	5073	Jebel Azourki (Jaz)	31.75847	−6.28826	2789	JX462119	JX462215	…	…	…	…	…
5111	5111	Jebel Azourki (Jaz)	31.75847	−6.28826	2789	JX462124	JX462217	…	…	…	…	…
6016	6816a	Outabati (Out)	32.17714	−5.33214	2441	JX462128	JX462221	JX461671	JX462037	JX485330	JX461853	JX461503
	6816b							JX461672	JX462038	JX485331	JX461854	JX461504
11754	11754a	Outabati (Out)	32.17714	−5.33214	2441	JX462140	JX462230	JX461551	JX461903	JX485350	JX461717	JX461375
	11754b							JX461552	JX461904	JX485351	JX461718	JX461376
11746	11746a	Outabati (Out)	32.17714	−5.33214	2441	JX462137	JX462228	JX461547	JX461899	JX485346	JX461713	JX461371
	11746b							JX461548	JX461900	JX485347	JX461713	JX461371
11743	11743a	Outabati (Out)	32.17714	−5.33214	2441	JX462135	JX462226	JX461543	JX461895	JX485342	JX461709	JX461367
	11743b							JX461544	JX461896	JX485343	JX461710	JX461368
11717	11717a	Outabati (Out)	32.17714	−5.33214	2441	JX462130	JX462222	JX461533	JX461885	JX485332	JX461699	JX461357
	11717b							JX461534	JX461886	JX485333	JX461700	JX461358
11755	11755a	Outabati (Out)	32.17714	−5.33214	2441	JX462139	JX462231	JX461553	JX461905	JX485352	JX461719	JX461377
	11755b							JX461554	JX461906	JX485353	JX461720	JX461378
11727	11727a	Outabati (Out)	32.17714	−5.33214	2441	JX462131	JX462232	JX461535	JX461887	JX485334	JX461701	JX461359
	11727b							JX461536	JX461888	JX485335	JX461702	JX461360
11752	11752a	Outabati (Out)	32.17714	−5.33214	2441	JX462138	JX462229	JX461549	JX461901	JX485348	JX461715	JX461373
	11752b							JX461550	JX461902	JX485349	JX461716	JX461374
6643	6643	Outabati (Out)	32.17714	−5.33214	2441	JX462129	JX462220	…	…	…	…	…
11741	11741a	Outabati (Out)	32.17714	−5.33214	2441	JX462134	JX462225	JX461541	JX461893	JX485340	JX461707	JX461365
	11741b							JX461542	JX461894	JX485341	JX461708	JX461366
11734	11734a	Outabati (Out)	32.17714	−5.33214	2441	JX462133	JX462224	JX461539	JX461891	JX485338	JX461705	JX461363
	11734b							JX461540	JX461892	JX485339	JX461706	JX461364
11745	11745a	Outabati (Out)	32.17714	−5.33214	2441	JX462136	JX462227	JX461545	JX461897	JX485344	HX461711	JX461369
	11745b							JX461546	JX461898	JX485345	HX461712	JX461370
11733	11733a	Outabati (Out)	32.17714	−5.33214	2441	JX462132	JX462223	JX461537	JX461889	JX485336	JX461703	JX461361
	11733b							JX461538	JX461890	JX485337	JX461704	JX461361
6639	6639	Outabati (Out)	32.17714	−5.33214	2441	JX462127	JX462219	…	…	…	…	…
3865	3865a	Toubkal (Tou)	31.09415	−7.91367	2600	JX462142	JX462236	JX461627	JX461981	JX485360	JX461797	JX4614513
	3865b							JX461628	JX461982	JX485361	JX461798	JX4614514
13276	13276a	Toubkal (Tou)	31.09415	−7.91367	2600	JX462143	JX462237	…	JX461909	JX485362	JX461723	JX461381
	13276b							…	JX461910	JX485363	JX461724	JX461382
5090	5090a	Jebel Awlime (JAw)	30.81708	−8.86298	2967	JX46244	JX462234	JX461651	JX462011	JX485354	JX461827	JX461481
	5090b							JX461652	JX462012	JX485355	JX4618288	JX461482
13179	13179a	Jebel Awlime (JAw)	30.81708	−8.86298	2967	JX462146	JX462235	JX461555	JX461907	JX485358	JX461721	JX461379
	13179b							JX461556	JX461908	JX485359	JX461722	JX461380
5123	5123a	Jebel Awlime (JAw)	30.81708	−8.86298	2967	JX462145	JX462233	JX461663	JX462025	JX485356	JX461841	JX461493
	5123b							JX461664	JX462026	JX485357	JX461842	JX461494

Genomic DNA was extracted from ethanol-preserved tissue samples using standard high-salt protocols [80]. A total of 89 specimens of *Atlantolacerta andreanskyi* plus three outgroups (*Podarcis hispanica*, *Podarcis carbonelli* and *Podarcis bocagei*) were sequenced for two mitochondrial regions: partial *12S rRNA* (*12S*) and partial *NADH dehydrogenase 4* (*ND4*) and flanking *tRNA (tRNA-His*) and 77 specimens for five nuclear gene fragments, *recombination-activating gene 1* (*RAG1*), *acetylcholinergic receptor M4* (*ACM4*), *melanocortin receptor 1* (*MC1R*), *oocyte maturation factor Mos* (*C-MOS*) and *phosducin* (*PDC*). Primers used for both amplification and sequencing were: 12Sa and 12Sb [81] for the *12S* following the PCR conditions described in Harris and Arnold [82], ND4 and Leu for *ND4 + tRNA-His*, PCR conditions described in Arévalo *et al.*[83]; L2408 and H2920 for *RAG1* following the PCR conditions from Vidal and Hedges [84]; tg-F and tg-R [85] for *ACM4* with PCR conditions following Gamble *et al.*[86]; MC1RF and MC1RR for *MC1R* following PCR conditions described in Pinho *et al.*[87]; Lsc1 and Lsc2 for *C-MOS* following the PCR conditions from Godinho *et al.*[88]; and PHOF2 and PHOF1 for *PDC*, following PCR conditions described in Bauer *et al.*[89]. PCRs were carried out in 25 μl volumes, containing 5.0 μl of 10 reaction Buffer, 2.0 mM of MgCl2, 0.5 mM each dNTP, 0.2 μM each primer, 1 U of Taq DNA polymerase (Invitrogen), and approximately 100 ng of template DNA. Finally, PCR products were purified using exosap IT and the resulting amplified fragments were sequenced on an Applied Biosystem DNA Sequencing Apparatus. Chromatographs were checked manually, assembled and edited using Bioedit 7.0.1 [90]. Sequences were aligned for each gene independently using the online version of MAFFT v.6 [91] with default parameters (gap opening penalty = 1.53, gap extension = 0.0) and FFT-NS-1 algorithm. Coding gene fragments (*ND4*, *C-MOS*, *ACM4*, *RAG1*, *PDC* and *MC1R*) were translated into amino acids and no stop codons were observed, suggesting that the sequences were all functional. Heterozygous individuals were identified based on the presence of two peaks of approximately equal height at a single nucleotide site. SEQPHASE [92] was used to convert the input files, and the software PHASE v2.1.1 to resolve phased haplotypes [93]. Default settings of PHASE were used except for phase probabilities that were set as ≥ 0.7 [94]. All polymorphic sites with a probability of < 0.7 were coded in both alleles with the appropriate IUPAC ambiguity code. Phased nuclear sequences were used for the structure analysis; networks and species tree analysis, and the unphased sequences for the phylogenetic analyses (see below). DnaSP [95] was used to calculate the number of haplotypes (h) and mutations (η). Mega v.3.0 [96] was used to estimate uncorrected *p*-distances and to obtain the number of variable and parsimony informative sites.

### Phylogenetic analyses

Phylogenetic analyses were performed using maximum likelihood (ML) and Bayesian (BI) methods. JModelTest [97] was used to select the most appropriate model of sequence evolution under the Akaike Information Criterion [98]. ML analyses were performed with RAxML v.7.0.4 [99] with 100 random addition replicates. A GTR + I + G model was used and parameters were estimated independently for each partition (by gene). Reliability of the ML tree was assessed by bootstrap analysis [100] including 1000 replications. Bayesian analyses were performed with MrBayes v.3.1.2 [101] with best fitting models applied to each partition by gene and all parameters unlinked across partitions. The models selected for the different partitions were: *12S*, GTR + I + G; *ND4*, GTR + G; *tRNA-His*, GTR + I + G; *ACM4*, HKY + I; *C-MOS*, GTR + I + G; *MC1R*, HKY + I + G; *PDC,* GTR + I + G; and *RAG1*, GTR + I. Two independent runs of 5x10^6^ generations were carried out, sampling at intervals of 1000 generations producing 5000 trees. Convergence and appropriate sampling were confirmed examining the standard deviation of the split frequencies between the two simultaneous runs and the Potential Scale Reduction Factor (PSRF) diagnostic. Burn-in was performed discarding the first 1250 trees of each run (25%) and a majority-rule consensus tree was generated from the remaining trees. In both ML and BI alignment gaps were treated as missing data and the nuclear gene sequences were not phased.

### Nuclear Networks

The genealogical relationships between the populations were assessed with haplotype networks for all the individual nuclear genes, constructed using statistical parsimony [102] implemented in the program TCS v 1.21 [103] with a connection limit of 95%. This analysis was made with the phased sequences. Haplotypes were colored taking into account the population of origin.

### Population structure – Clustering analyses

A model-based Bayesian clustering method was applied to all haplotypes using STRUCTURE v.2.3.2 [60,104,105]. In this analysis, individuals are probabilistically assigned to either a single cluster (the population of origin), or more than one cluster (if there is admixture). STRUCTURE was run with haplotype information from the nuclear fragments independently. We ran our data with the all parameters combinations between the Ancestry Model and the Allele Frequency Model to compare the results. The genetic structure was forced to vary from K = 2 to K = 10 clusters, the latter corresponding to the number of geographic populations sampled plus two. STRUCTURE ran for 550 000 steps, of which the first 50 000 were discarded as burn-in. For each value of K ten independent replicates of the Markov Chain Monte Carlo (MCMC) were conducted. To detect the true number of clusters (K) we followed the graphical methods and algorithms outlined in Evanno *et al.*[61], with the comparison of the average posterior probability values for K (log likelihood; ln L) using the online version, STRUCTURE HARVESTER v0.6.5 (available at: http://taylor0.biology.ucla.edu/struct_ harvest/, April 2011).

### Species tree, and divergence time estimates

Here we applied the coalescent-based species-tree approach implemented in STARBEAST [106] an extension of BEAST v1.6.1 [107] to test the origin and diversification patterns in *Atlantolacerta*, and to compare these results to those obtained from the ML and BI analyses of the concatenated dataset. This analysis needs *a priori* information regarding the species/populations delimitation and the species/populations assignation of the individuals in order to reconstruct the topology of the species tree. For this approach, we used the results obtained from previous clustering analyses to define the groups of individuals to be used as “species” (populations) in STARBEAST [106]. The clustering analysis supported the existence of six lineages, as Oukaimeden, Toubkal and J. Awlime were included in the same lineage.

All five nuclear gene fragments, *12S* and the fragment consistent of the *ND4* and flanking *tRNA-His* were included in the analyses as 7 independent partitions. The phased dataset was used for the nuclear loci.

The input file was formatted with the BEAUti utility included in the software package. We performed two independent runs of 1.5 x 10^8^ generations, sampling every 15 000 generations, from which 10% were discarded as burn-in. Models and prior specifications applied were as follows (otherwise by default): *12S* - GTR + G; *ND4* and *tRNA-His* - HKY + G; *MC1R* - HKY + I; *ACM4* - HKY + I; *C-MOS* - GTR + I + G; *RAG1* - HKY + I; *PDC* - GTR + I; Relaxed Uncorrelated Lognormal Clock (estimate); Yule process of speciation; random starting tree; alpha Uniform (0, 10).

For all analyses implemented in BEAST, convergence for all model parameters was assessed by examining trace plots and histograms in Tracer v1.5 [108] after obtaining an effective sample size (ESS) > 200. The initial 10% of samples were discarded as burn-in. Runs were combined using LogCombiner, and maximum credibility trees with divergence time means and 95% highest probability densities (HPDs) were produced using Tree Annotator (both part of the BEAST package). Trees were visualized using the software FigTree v1.3.1 [109].

Several studies have already calculated divergence rates for reptiles, and particularly for lacertids [2,15,49]. Pinho *et al*. [15] used well-known and dated independent geological events in the Aegean [110] to estimate a maximum and minimum mutation rate for the *ND4* mitochondrial fragment (and flanking *tRNA-His*) for the lacertid lizards of the genus *Podarcis* (0.0278 and 0.0174 mutation/site/million years, respectively). However, this was the only information available for our data, since we did not have any fossils or calibrations for nuclear markers. It is important to bear in mind that, in the absence of accurate calibration points in the phylogeny from external and independent data (fossil records, known biogeographic events, or paleoclimatic reconstructions) or as a result of the heterogeneity in the evolutionary rate between the calibrated and uncalibrated taxa, temporal estimates by means of molecular data could be a potential source of inference error, and, therefore, they should be treated with caution [111]. Despite the limitations of molecular clocks [111,112], divergence time estimates can still provide a proxy for the temporal window of evolutionary diversification in species groups of interest. Therefore and taking into account our data limitations and availability, we used BEAST v.1.6.1 [107] to estimate dates of the cladogenetic events using only *ND4* and flanking *tRNA-His*. We used a phylogeny pruned arbitrarily to include one representative from each of the major lineages uncovered with the concatenated analysis (6 specimens in total, we excluded J. Awlime population, because of the lack of support of the branch in previous analyses). This method excludes closely related terminal taxa because the Yule tree prior (see below) does not include a model of coalescence, which can complicate rate estimation for closely related sequences [113]. Analyses were run four times for 5x10^7^ generations with a sampling frequency of 10 000. Models and prior specifications applied were as follows (otherwise by default): GTR + G for *12S*; HKY + G for *ND4* and *tRNA-His*; HKY + I for *MC1R*; HKY + I for *ACM4;* GTR + G + I for *C-MOS*; HKY + I for *RAG1*; GTR + I for *PDC*; Relaxed Uncorrelated Lognormal Clock (estimate); Yule process of speciation; random starting tree; alpha Uniform (0, 10); ucld.mean of *ND4* Normal (initial value: 0.0226, mean: 0.0226, Stdev: 0.0031).

## Authors’ contributions

MB carried out the molecular laboratory work, analyzed the data and drafted a preliminary version of the manuscript. All authors participated in the conception and design of the study, collection of samples, writing and approval of the final manuscript.
